# Experiences of student nurses on role modelling of a therapeutic approach by registered nurses: Recommendations for improvement

**DOI:** 10.4102/curationis.v44i1.2168

**Published:** 2021-08-11

**Authors:** Esther A. Mpangane, Agnes Makhene, Hafisa Ally

**Affiliations:** 1 Department of Nursing, Faculty of Health Sciences, University of Johannesburg, Johannesburg, South Africa

**Keywords:** experiences, student nurses, role modelling, therapeutic approach, registered nurses

## Abstract

**Background:**

A therapeutic approach involves portraying the attributes of being polite towards fellow human beings and patients, respecting them irrespective of their circumstances and having sympathy and compassion for them. Knowledge of therapeutic approach is the initial step towards gaining patients’ trust and developing student nurses’ communication with patients; however, theoretical knowledge alone may not increase application in practice. Role modelling of a therapeutic approach increases patient care satisfaction and enables student nurses to therapeutically communicate with patients, colleagues and all other staff members. The most appropriate way for student nurses to learn what it means to portray therapeutic approach is seeing registered nurses’ role modelling it.

**Objectives:**

To make recommendations for the enhancement of role modelling of therapeutic approach by registered nurses for student nurses at a regional public hospital.

**Method:**

A qualitative, exploratory, descriptive, phenomenological and contextual design was used. Three focus group interviews were conducted. Data were analysed using Giorgi’s descriptive phenomenological method. Measures to ensure trustworthiness and ethical principles were applied throughout the research.

**Results:**

One central theme with three main themes and related sub-themes indicated that student nurses had negative experiences on role modelling of therapeutic approach owing to registered nurses’ non-therapeutic communication, lack of professionalism and ethical conduct as well as poor quality patient care. However, only the recommendations seeking to address the experiences of non-therapeutic communication which included negative attitudes of registered nurses towards patients, lack of provision of patient information, poor handling of patients’ complaints as well as racial discrimination re-handling of patients’ complaints will be highlighted.

**Conclusion:**

The majority of student nurses had negative experiences on role modelling of therapeutic approach by registered nurses. They needed registered nurses to improve their approach towards patients. It is expected that the implementation of the recommendations will enhance therapeutic approach to patients.

## Introduction

A therapeutic approach involves portraying the attributes of being polite towards fellow human beings and patients, respecting them irrespective of their circumstances and having sympathy and compassion for them (Felstead 2013:223). Not all the attributes of a therapeutic approach can be taught in the classroom, but student nurses will just pick up some cues of behaviours from registered nurses during their interaction with patients, intuit and internalise them (Askola et al. 2016:164). Knowledge of therapeutic approach is the initial step towards gaining patients’ trust and developing student nurses’ communication with patients; however, theoretical knowledge alone may not increase application in practice. Cunze (2016:63) mentioned that it is important that student nurses are taught and developed on how to therapeutically communicate with patients whilst student nurses placed in the clinical settings through role modelling. The absence of role modelling of therapeutic approach by registered nurses may lead to student nurses’ confusion between therapeutic approach theory learned in the classroom and which they are confronted with and are expected to live out in clinical practice (Cunze 2016:8).

A therapeutic approach is one of the most effective means of communication that the registered nurses have to use for building trust with the patients and providing opportunities for student nurses to communicate with patients effectively. However, their approach towards patients has been always publicly criticised by media (Khaya FM 2016). Developing therapeutic approach through role modelling for student nurses should be the registered nurses concern in the clinical setting to enable them to improve their communication skills (Cunze 2016:63). The attributes of therapeutic approach can be understood and appreciated by student nurses through role modelling during interaction with patients (Lovan & Wilson 2012:30). However, role modelling the attributes of therapeutic approach only when with student nurses can be questioned because planned behaviour gives a false impression. Student nurses will not be observing an honest reflection of a therapeutic approach if the registered nurses are aware that they are being watched (Felstead 2013:223). This view is supported by Begum and Slavin (2012:332), who added that role modelling of a therapeutic approach should just take place spontaneously as part of the hidden curriculum for student nurses to observe and learn a genuine approach. Noddings (2010:394) noted that successful practice of therapeutic approach depends on the role model and the observer’s experience. Therefore, registered nurses’ approach during interaction with patients should not be underestimated as student nurses will watch, copy and imitate them.

Teaching of the concept of therapeutic approach happens in the classroom, but it is best integrated with the skills learned in the clinical placement setting where integration of theory and practice takes place (Cunze 2016:63). De Swardt (2012:57) understood that therapeutic approach experiences are difficult to simulate successful in the simulation laboratory setting, except in the real-life clinical setting whereby student nurses will observe and imitate the approach copied from registered nurses as their role models. In the classroom settings, a perfect scenario for therapeutic approach is sketched, which is not a reflective of the real-life situation in the clinical placement setting (Waxman 2010:57).

Coupled with the above difficulty is the registered nurses’ questionable approach towards patients, which could be contradictory to the theoretical approach learned in the classroom. The student nurses’ contradictory experiences have caused many of them to question whether the attributes of the therapeutic approach extend beyond the theoretical confinements of the classroom into the clinical context where they ought to be role modelled by registered nurses in a practical manner.

Cleary, Deacon and Hunt (2011:7) mentioned that although teaching of a therapeutic approach and role modelling of the attributes of a therapeutic approach are frequently in isolation from one another, it is only through role modelling that it can be understood and appreciated by student nurses. In addition, Felstead (2013:223) affirmed that role modelling of a therapeutic approach for student nurses ‘cannot be taught, but can be caught’ by following examples of registered nurses. De Swardt (2012:57) mentioned that the most appropriate way for students to learn a therapeutic approach is to see it in action during their placement in the clinical setting. Cunze (2016:175) supported that ‘it is one thing to be taught a therapeutic approach in the classroom, but it is another thing to experience role modelling of it at clinical settings’, and that it is easier to imitate behaviour than a spoken word. Undoubtedly, role modelling of a therapeutic approach by registered nurses for student nurses is needed in developing their approach towards patients. Askola et al. (2016:164) added that the art of a therapeutic approach which student nurses need to exhibit should be imparted to them whilst they are still novices in the nursing profession through role modelling, as such approach may make a difference in student nurses communication with patients. The absence of role modelling of a therapeutic approach by registered nurses may lead to student nurses’ confusion between nursing professional approach learned in the classroom and those which they are confronted with and expected to live out in clinical practice (Cunze 2016:8).

A reflective dialogue between clinical coordinators and student nurses during clinical accompaniment about their experiences on role modelling of a therapeutic approach revealed a gap between the theoretical content in relation to the practical experiences witnessed in the clinical placement.

## Definition of key concepts

### Experiences

Experience refers to an activity that involves gaining knowledge by being personally involved in an event, situation or circumstance (Burns & Grove 2011:35). It is a knowledge gained through practice and observation (Oxford Advanced Learners’ Dictionary 2010:89). In this study, experience refers to the student nurses’ views, occurrences and impressions on role modelling of a therapeutic approach.

### Student nurse

Student nurse is a person registered as such in terms of *section 32(1) of the Nursing Act No.33 of 2005*, undergoing education or training in nursing. In this study, student nurse refers to a person who is registered in the Regulation (R683) nursing programme, is in level two of training and placed at a regional public hospital for clinical practice.

### Role modelling

Role modelling is defined as the ability of registered nurses to lead by example and influence student nurses’ ability to practise the therapeutic approach towards patients (Felstead 2013:223). In this study, registered nurses will teach student nurses the practice of therapeutic approach in the clinical setting through role modelling whilst student nurses observe and imitate them (Burgess, Oates & Goulston 2016:134).

### Therapeutic approach

A therapeutic approach refers to the attributes that involve being polite and friendly towards fellow human beings, being there for the patients, respecting patients irrespective of their circumstances and having sympathy and compassion towards the patients (Rosenberg 2011:6). In this study, a therapeutic approach entails the demonstration of gestures such as active listening, trusting, respecting, empathetic, offering verbal reassurances, responding to patients and families’ concerns, genuine interest and engagement through role modelling.

## Methods and design

In this study, a qualitative, exploratory, descriptive, phenomenological and contextual design was used to describe and understand student nurses’ experiences on role modelling of therapeutic approach by registered nurses.

### Population and sampling

The target population of all student nurses registered with the South African Nursing Council for the Bridging Course programme of enrolled nurses leading to registration as a general nurse (R683) (*Nursing Act no. 33 of 2005*), having placed at a regional public hospital for clinical ward management learning outcomes and willing to participate voluntarily in the study were the inclusion criteria. The purposive sampling method was used for data collection. A total of three focus group interviews were conducted until data saturation was reached.

### Data collection

In this study, the focus group interviews method of data collection was used. An independent interviewer who has a master’s degree in psychiatric nursing and also has specialised interviewing skills in qualitative research conducted the focus group interviews. This was necessary in order to prevent bias as the researcher is the clinical co-coordinator of the level group of the participants. Each interview lasted for 30 min – 45 min. The researcher arranged six chairs in a circle seating to allow eye contact, free communication between the participants and the interviewer for the researcher to observe any non-verbal cues necessary to add to the data. The two central questions asked to the participants were: ‘what are your experiences on role modelling of therapeutic approach by registered nurses, and; what can be performed to enhance role modelling of therapeutic approach for student nurses?’ Subsequent questions were determined by the student nurses’ responses to the central question. The interviews were recorded using an audio tape recorder with the permission obtained from the participants who agreed to participate in the study.

### Data analysis

Giorgi’s descriptive phenomenological five-step methods were used (Giorgi’s & Giorgi 2003:80), which entailed the researcher bracketing her previous presuppositions, cultural, theoretical or experiential knowledge and attempting to look at the data with clear mind, reading through the interviews several times to attain a sense of the whole, repeatedly reading the transcripts and identified the separate meaning entities, reviewing the identified meaning units and themes against the purpose of the study and forming of important non-conflicting themes together in a descriptive statement. The independent coder analysed the transcriptions of the interviews and compared it with the researcher’s coding decision for coding consistency. A consensus discussion between the independent coder and the researcher was conducted.

### Ethical considerations

Ethical approval to conduct the study was obtained from the Faculty of Health Sciences Research Ethics Committee, University of Johannesburg, reference number: REC-01-143-2017 as stipulated in the *National Health Act* No. 61 of 2003. The researcher adhered to the ethical principles of autonomy and respect for persons, anonymity and confidentiality, non-maleficence and justice as indicated by Dhai and McQuoid-Mason (2011:166–179).

Autonomy: The participants were given an explanation and information letter about the proposed study, which included information about their right to voluntary participation, right to withdraw without penalty, a request to sign the informed consent after reading and understanding the letter, request to record interview as well as their reassurance to confidentiality, anonymity and privacy. Beneficence and non-maleficence: there were no direct benefits for participating in the study, but recommendations were developed and provided to student nurses to ensure that no harm was experienced by the participants during the study. Justice: the researcher adhered to the principle by ensuring that there was fair recruitment in the process of selection of the participants.

## Results

One central theme: Student nurses experienced role modelling of therapeutic approach negatively, three main themes: (1) non-therapeutic communication, (2) lack of professionalism and ethical conduct of registered nurses as well as (3) poor quality patient care rendered by registered nurses and related sub-themes emerged from data analysis. However, only the recommendations pertaining to sub-themes of Theme 1: experiences of non-therapeutic communication will be described. These include: negative attitudes of registered nurses towards patients, lack of provision of patient information, poor handling of patients’ complaints, racial discrimination: re-handling of patients’ complaints.

### Theme 1: Experienced non-therapeutic communication

The experiences of non-therapeutic communication demoralise student nurses’ desire to learn and practise the therapeutic approach. Communication that humiliates patients is regarded by participants as negative experience and creates effects such as confusion in the learning experiences of student nurses. Non-therapeutic communication was regarded as shouting at the patients and being unapproachable. Participants described how the registered nurse shouted at the patients in front of other patients and staff members:

‘The registered nurse who was sister in charge shouted at the patient, forgetting that the other patients are listening; it is very bad.’ (P2, group 2, 12 June 2018)

‘When there is high workload … shooo!!! They [*registered nurses*] will shout at patients without even considering their age.’ (P2, group 2, 12 June 2018)

‘When the patient explained to her [*registered nurse*] that she stopped menstruating yesterday, the registered nurse started shouting at the patient in front of other patients.’ (P4, group 1, 30 Apr. 2018)

Non-therapeutic communication of registered nurses is a challenge because patients are reporting to be scolded by nurses when they ask to be attended to. The findings of the study conducted by Ndou, Maputle and Risenga (2015:4) revealed that the shouting of patients by the healthcare providers and sending them home without help not only compromise nurse-patient therapeutic relationship, but also hinder student nurses’ positive experiences on role modelling of therapeutic approach. Ajani and Moez (2011:3928) added that the effect of non-therapeutic communication approach is that it minimises opportunities for training and development of student nurses on therapeutic communication approach.

The non-therapeutic communication approach of registered nurses towards patients cannot be taken-for-granted as it impacts patients care and student nurses’ benefits of developing the art of therapeutic communication with patients. Participants (P) in this study expressed the need for the implementation of the Peer Review Team (PRT) for registered nurses.

‘I suggest that the management initiate and implement peer and self-evaluation on therapeutic communication approach of the registered nurses towards the patients in the unit and provide feedback.’ (P7, group 3, 30 Apr. 2018)

Nel, Ally and Dlamini (2016:11) concurred that managers should consider implementing a PRT which would contribute to holding registered nurses and team members accountable for their non-therapeutic approach. In addition, Dubi, Becker and Tekian (2015:534) affirmed that feedback on peer and self-evaluation of nurses is essential for the motivation of registered nurses to improve role modelling of therapeutic approach for student nurses in the clinical setting.

### Sub-theme 1.1: Negative attitude of registered nurses towards patients

Nursing is a caring profession characterised by care and compassion. The expectation is that nurses should have a caring attitude in order to render holistic nursing care. Patients, on the other hand, assume that they will be treated with such caring attitude. According to participants, negative attitude of registered nurses is characterised by lack of demonstration of kindness, compassion for patients and not interested in teaching and learning of student nurses in the clinical placement settings:

‘When the patient reports to the registered nurse that the vacoliter is empty and it is causing pain, the registered nurse will say “they will change it,” leaving the patient unattended in pain.’ (P4, group 1, 30 Apr. 2018)

‘The therapeutic approach is poor because you find that the doctor has prescribed pethidine for the patient post operatively, the registered nurse will say “don’t give,” where will she get it when she is at home, she will get used to the drugs and leaving the patient in pain. What if it was me feeling pain and the doctor has prescribed something for me to relieve that pain and then somebody is not giving me; it does not sit well with me’. [*sad*] (P1, group 2, 12 June 2018)

Participants also described that being disrespectful towards patients and treating them like they are not human beings have a negative impact on student nurses’ current and future positive experiences on role modelling of therapeutic approach:

‘She [*registered nurse*] just talk the way she want without showing some respect, and even patients, when they see her or him, they make comments saying “oh…u khona ke namhlanje” … [*meaning she is here today*].’ (P1, group 1, 30 Apr. 2018)

Participants mentioned that nursing managers should encourage and motivate registered nurses to self-introspect their approach towards patients which has been always criticised publicly by media reports. Management support and motivation would shape the positive attitude of registered nurses towards patients and improve the therapeutic approach observed by the student nurses:

‘The OM [*Operational Manager*] must remind the registered nurses to always do self-introspection of their communication approach towards the patients, relatives and other staff members and rate themselves as to whether it was negative or positive.’ (P7, group 3, 12 June 2018)

‘Management must support registered nurses by motivating them, but not rewarding those registered nurses who are found to be portraying positive attitudes when communicating with patients. Just acknowledge.’ (P11, 12 June 2018)

Sundler et al. (2014:665) asserted that time for reflection should be allocated to allow registered nurses to reflect on their attitude, why it did happen and how they are planning to improve those negative attitudes. Such reflection time would be more valuable for student nurses to also reflect on their own attitude towards patients, peers and other staff members.

### Sub-theme 1.2: Lack of provision of patients’ information

Participants articulated that lack of provision of patients’ information affected student nurses’ experiences on role modelling of therapeutic approach. Participants described lack of provision of patients’ information as patients being discharged without knowledge of their diagnosis:

‘Batho Pele Principles are not applied. Patients are not getting enough information from the registered nurses. Patients are discharged without knowing the diagnosis they were admitted for such as Asthma, COPD [*chronic obstructive pulmonary disease*], and when they ask nurses, they will just say ‘u ne Asthma [*meaning … you have asthma*], no further explanation, nothing more given to the patient at all.’ (P11, group 1, 30 Apr. 2018)

They further alluded that patients are blamed and shouted for not cooperating in the nursing intervention regime, whereas registered nurses did not give them health education with regard to what is expected from them (patients):

‘When the patient is getting intravenous treatment and was not informed about the expectations from her side regarding what to do when the vacoliter is empty, instead, some of the patients will just remove the IV [*intravenous*] line, and the registered nurses will shout at the patient as if she has told the patient that she mustn’t remove it.’ (P7, group 3, 12 June 2018)

Participants believe that therapeutic approach would improve when patients are given information regarding their diagnosis and interventions. Participants said the following sentiments:

‘I think patients should be given information on discharge regarding their diagnosis and emphasise what they are expected to do in order to help in the intervention plan and increase compliance. Patients should be informed verbally of their next appointment date and be given appointment card with date written on it before the patient leaves the hospital.’ (P6, group 2, 12 June 2018)

‘Registered nurses must be given in-service training on the importance of giving patient health education.’ (P3, group 2, 12 June 2018)

When patients are provided with information as part of role modelling of therapeutic approach, and fully understood their conditions, the number of readmissions will decrease whilst improving compliance (Clwyd & Hart 2013:15), and student nurses would, in turn, feel comfortable enough to communicate health education plan with patients.

### Sub-theme 1.3: Poor handling of patients’ complaints

Participants experienced that poor handling of patients’ complaints by registered nurses has a great negative impact on the student nurses’ experiences on role modelling of therapeutic approach as patients also become dissatisfied. The participants reported that the non-attendance of patients’ complaints and failure of the registered nurses to handle them promptly according to the complaint management protocol discourage patients from lodging complaints. The participants further articulated that patients perceive the registered nurses as not caring because they do not communicate what is being performed to their lodged complaints:

‘Some patients do not want to use the suggestion box. They are negative about the suggestion box because they say, ‘we wrote and put in the box but nothing is being done, maybe they just throw it away without reading and attending to what is inside.’ (P4, group 2, 12 June 2018)

‘I think in terms of these suggestions that have been written by the patients and families. What I have noticed is that they [*registered nurses*] take long to give them feedback.’ (P11, group 3, 12 June 2018)

‘Patients complain that because every time we write something, nothing is being done, the situation remains like that.’ (P6, group 2, 12 June 2018)

Clwyd and Hart (2013:21) in their study on patient satisfaction found that many patients who complain felt that no attempts had been made to understand and address their complaints.

Participants understood that there should be a policy or guideline stipulating that patients’ complaint be handled by an external committee for prompt communication of the outcomes of the complaints and improved patients’ satisfaction:

‘There must be a committee formed within the hospital which will advocate for the patients and the committee must at least have community representatives of different races. Patients’ complaints must be reported to that committee.’ (P6, group 2, 12 June 2018)

‘I think the other way to attend to these complaints accordingly is when managers are hands off the complaints; only community representatives are handling those complaints because they will not be biased because one day it will be them or their family members who are approached negatively by registered nurses.’ (P1, group 1, 12 June 2018)

Handling of patients’ complaints locally than patients reporting their unresolved complaints to the media and to the Health Service Ombudsman was understood by participants as important to prevent miscommunication that might potentially be an obstacle for student experiences on therapeutic approach. In the same vein, Patole (2015:46) concurred that the effective communication and transparency in handling of patients’ complaints will enable student nurses to experience role modelling of therapeutic approach positively.

### Sub-theme 1.4: Racial discrimination: Re-handling of patients’ complaints

Registered nurses took an oath to treat all patients equally, and yet not all patients are treated equally well. The discrimination may significantly impact the student nurses’ learning of communication and the quality of patient care. Participants expressed having experienced racial discrimination by registered nurses when handling patients’ complaints. Participants reported that there is a delay in attendance of patients’ complaints laid by black patients’ as compared to their whites’ counterparts, which left the student nurses having concern of learning therapeutic approach in such a discriminatory clinical setting:

‘Once a white person laid a complaint, it will be attended to as soon as possible, but if it is the black person, there will be no concern that much although it will be attended to.’ (P3, group 2, 12 June 2018)

‘Patients are normally complaining, but once white and African person laid complaint, the complaint that has been laid by a white person will be attended as soon as possible compared to the complaint that was laid by an African person. Patients’ are not equally treated by the registered nurses.’ (P6, group 2, 12 June 2018)

Clwyd and Hart (2013:9) in their study on patient satisfaction found that registered nurses practice in a discriminatory way in handling the patients’ complaints procedure. Geyer (2013:54) asserted that a registered nurse in her professional role is responsible for treating all patients equally without portraying some form of discrimination. However, to date, some nurses still exhibit racial discrimination towards black patients (Durrheim et al. 2011:263). Participants reported that handling of patients’ complaints equally without displaying discrimination to other racial colour would be essential for student nurses to learn and improve their communication skills:

‘I think if we can treat all patients equally irrespective of the colour of their skin.’ (P2, group 2, 12 June 2018)

‘The management must treat all complaints equally, they must not check and compare if it is Van Wyk or Mnisi who laid the complaints, they must treat the way they should.’ (P1, group 1, 30 Apr. 2018)

Clwyd and Hart (2013:21) reinforced that nurses must adopt ‘a light touch’ approach than a full formal investigation of patients’ complaints.

## Discussion

The objective of this study was to make recommendations for the improvement of role modelling of therapeutic approach by registered nurses for student nurses. The study revealed that non-therapeutic communication of registered nurses was characterised by negative attitude towards patients, lack of provision of patient information and poor handling of patients’ complaints. They also experienced racial discrimination; re-handling of patients’ complaints which was also seen as barrier or a negative experience on therapeutic approach and the learning of communication skills in the clinical settings. This is consistent with the findings by Calson and Idvall (2014:1130) that the non-therapeutic communication approach of registered nurses results in student nurses taking shortcuts to complete their tasks quickly and missing opportunities which would have allowed them to learn a therapeutic communication approach.

Therefore, the need for the implementation of PRT for registered nurses to ensure improvement of therapeutic approach, and that student nurses apply such approach when communicating with patients and the entire staff members. Hospital management should provide registered nurses feedback on their peer and self-evaluation to assist them to improve on their non-therapeutic communication.

Participants experienced that the portray of negative attitude towards patients is a form of undermining the student nurses’ therapeutic approach knowledge learned in the classroom. The reasons for negative attitude that were reported in the findings of this study were, amongst others, registered nurses’ lack of respect for patients, treating them like they are not human beings and unfriendliness which are barriers to a therapeutic approach and intolerable by both patients and student nurses. Similar experiences have been reported in previous studies wherein nurses do not introduce themselves to patients and they are rude to them (Tomas 2017:25). In the same vein, Clwyd and Hart (2013:16) concurred that registered nurses frequently did not make time to speak to patients in a friendly and concerned way, which is not what patients and student nurses expect from nurses providing care. Banaser, Stoddart and Cunningham (2017:5) found that unfriendliness and impoliteness of registered nurses in the clinical setting decrease the student nurses’ ability to have a therapeutic communication during their interaction with patients. The findings suggest that management support and motivation for registered nurses identified to be portraying negative attitudes are ideal to ensure improved attitude as expected in the nursing profession. Equally, registered nurses should reflect on their attitude, why they portray negative attitude towards patients and how they are planning to improve those negative attitudes that exist in the clinical setting. Such reflection time would be more valuable for student nurses to also reflect on their own attitude towards patients, peers and other staff members.

The importance of providing patients with health information in the form of education, therefore, becomes essential as it has indicated that student nurses experienced lack of provision of patients’ information during their clinical placement. Patients are being discharged without knowledge of their diagnosis, blamed and shouted for not cooperating in the nursing intervention regime, whereas registered nurses did not give them health education with regard to what is expected from them as patients. Student nurses are also blamed when things go wrong in the unit because they were not orientated as newcomers and do not know the routine, policies and procedures. The findings of the study carried out by Clwyd and Hart (2013:15) on patient satisfaction revealed that lack of provision of patient information has led to patients having re-admitted for the same conditions several times. Oyetunde and Akinmeye (2015:50) asserted that patient poor participation in their treatment regime is mainly because of lack of provision of information and not actively engaged in their care.

Not being exposed to a positive clinical learning where patients are provided with health education, student nurses would experience struggle to teach patients and peers without relevant information. This dynamic created a condition that constrained them from achieving competency in learning outcomes on provision of patient health education. The Batho Pele Principle of right to information (2000) stipulated that patients should be given full accurate information about the service they are entitled to receive. This is essential for student nurses learning their therapeutic communication skills with patients, especially for the novice student nurses at clinical placement setting who may be unsure of their therapeutic communication skills. Participants strongly recommended that patient’s treatment plan should be communicated with them and all those who are involved with the patient. As a result, student nurses would learn the approachable communication with the patients and the entire team members in the clinical practice environment.

The findings also indicated that student nurses experienced poor handling of patients’ complaints. Patients are dissatisfied with how their written complaints are being handled by registered nurses. The participants expressed that the non-attendance of patients’ complaints and failure of the registered nurses to handle them promptly according to the complaint management protocol discourage patients from lodging complaints. It is also associated with negative experiences on role modelling of therapeutic approach. Many patients who complain felt that no attempts had been made to understand and address their complaints (Clwyd & Hart 2013:21). Delays in processing, resolving and providing patients feedback regarding the outcomes of their complaints create a huge frustration in patients and families, as they receive no explanation for the reasons of delays and are kept uninformed about where their complaint had reached in the complaint system. Providing patients’ feedback of the outcomes of their complaints creates a positive relationship and trust between patients and all other staff members (Yoo & Park 2015:170). Handling of patients’ complaints at managerial level with the inclusion of external committee will bring about improved patients’ satisfaction. Handling of patients’ complaints locally as opposed to patients reporting their unresolved complaints to the media and to the Health Service Ombudsman should be considered as another intervention for improving therapeutic approach. Thus, ‘a light tough investigation approach’ was recommended. Participants strongly recommend that hospital management and policy makers should develop and implement effective policies which will clearly define the role and responsibility of the registered nurses regarding handling of patients’ complaints.

The findings also indicated racial discrimination: re-handling of patients’ complaints. Registered nurses are seen to be portraying a form of delay when attending patients’ complaints laid by black patients’ as compared to their whites’ counterparts. Furthermore, registered nurses discriminate patients by allowing certain race of patients to use wrong complaints reporting channels whilst other races do not have the luxury to such channels. Similar experiences of racial discrimination have been reported in previous studies. A registered nurse in her professional role is responsible for treating all patients equally without portraying some form of discrimination (Geyer 2013:54). However, to date, some nurses still exhibit racial discrimination towards black patients (Durrheim et al. 2011:263). Therefore, admitting and talking openly about existing racial discriminatory dynamics regarding handling of patients’ complaints are considered vital for positive experiences of student nurses on therapeutic approach. The participants lamented that the approach to investigate and handle patients’ complaints should consider the seriousness of the complaint lodged and not the racial colour.

## Recommendations

The following recommendations are made because of student nurses’ negative experiences on the role modelling of therapeutic approach by registered nurses, directed towards nursing practice, nursing education and nursing research: Registered nurses should *inter alia* be encouraged to conduct peer and self-evaluation on their attitude towards patients to determine areas of improvement of therapeutic approach, and be provided with the feedback thereof. Registered nurses who portray positive attitude towards patients should not be rewarded, but be acknowledged because rewarding may displace the genuine positive attitude which student nurses are expected to imitate. It is furthermore recommended that registered nurses should give patients full and accurate information regarding their health problems, and thus improve the therapeutic communication approach. Reinforcement of nurse managers provides registered nurses in-service training on the importance of providing patients’ health education. It is strongly recommended that hospital management and policy makers should develop and implement effective policies which will clearly define the role and responsibility of the registered nurses regarding handling of patients’ complaints. Patients’ complaints are handled by an independent committee or board formed by community members with the registered nurses serving as overseers in order to facilitate a harmonious way of handling of patients’ complaints. Hospital managers should consider handling of patients’ complaints locally than patients reporting their unresolved complaints to the media and Health Service Ombudsman. Thus, adopting ‘a light touch’ approach is needed than a full formal investigation of patients’ complaints. Admitting and talking in a dialogic conversation platform openly about the existing racial discriminatory dynamics regarding handling of patients’ complaints as a strategy to improve therapeutic approach are welcomed. The approach to investigate and handle patients’ complaints’, however, should depend on the seriousness of the complaint lodged and not on the racial colour.

The researcher recommends that further studies on the registered nurses’ perceptions regarding their own role modelling of therapeutic approach be conducted. This will present a more holistic perspective on role modelling of therapeutic approach at regional public hospitals.

## Limitations of the study

The study was conducted in one regional public hospital in Gauteng province. Different experiences may have been described by student nurses in other regional public hospitals, which might impact the transferability of the findings. Some participants were not present for focus group interviews because they were on night duty. It would have been possible to base this study on a much wider range of experiences if all the participants were present.

## Conclusion

It was evident from the study that student nurses experienced role modelling of therapeutic approach negatively which had impact on their approach towards patients and the entire staff members owing to registered nurses’ non-therapeutic communication, negative attitude towards patients, poor handling of patients’ complaints and portraying of racial discrimination in re-handling patients’ complaints. Recommendations for improvement were outlined so that registered nurses could be more approachable towards patients, and change the nurses’ negative approach towards patients which has been overly criticised on different media platforms. These recommendations will also ensure that student nurses feel more inspired and motivated to imitate such therapeutic approach during their placement in the clinical settings.
